# Injectable hydrogels for cartilage and bone tissue engineering

**DOI:** 10.1038/boneres.2017.14

**Published:** 2017-05-30

**Authors:** Mei Liu, Xin Zeng, Chao Ma, Huan Yi, Zeeshan Ali, Xianbo Mou, Song Li, Yan Deng, Nongyue He

**Affiliations:** 1 State Key Laboratory of Bioelectronics, School of Biological Science and Medical Engineering, Southeast University, Nanjing, PR China; 2 Nanjing Maternity and Child Health Care Hospital, Nanjing, PR China; 3 School of Applied Chemistry and Biotechnology, Shenzhen Polytechnic, Shenzhen, PR China; 4 School of Chemistry and Chemical Engineering, Southeast University, Nanjing, PR China; 5 Hunan Key Laboratory of Green Chemistry and Application of Biological Nanotechnology, Hunan University of Technology, Zhuzhou, PR China

## Abstract

Tissue engineering has become a promising strategy for repairing damaged cartilage and bone tissue. Among the scaffolds for tissue-engineering applications, injectable hydrogels have demonstrated great potential for use as three-dimensional cell culture scaffolds in cartilage and bone tissue engineering, owing to their high water content, similarity to the natural extracellular matrix (ECM), porous framework for cell transplantation and proliferation, minimal invasive properties, and ability to match irregular defects. In this review, we describe the selection of appropriate biomaterials and fabrication methods to prepare novel injectable hydrogels for cartilage and bone tissue engineering. In addition, the biology of cartilage and the bony ECM is also summarized. Finally, future perspectives for injectable hydrogels in cartilage and bone tissue engineering are discussed.

## Introduction

Cartilage and subchondral bone damage can be caused by a variety of conditions, such as trauma, arthritis, and sports-related injuries.^1,2, 3,4^ It has been reported that 60% of patients examined by knee arthroscopy exhibit cartilage damage, and ~15% of people over 60 years old have some clinical symptoms of such damage.^5,6^ In particular, the self-healing of damaged cartilage is limited, owing to its lack of vascularization, innervation, lymphatic networks, and progenitor cells.^6,7,8,9,10,11,12^ For bone tissue, despite its high vascularization, commonly used techniques for repair, such as autografting and allografting, are limited because of risks of donor-site morbidity, potential infection, and a high nonunion rate with host tissues.^13,14,15,16,17^ Bone defects are one of the leading causes of morbidity and disability in elderly patients.^18^ Medical restoration of the damaged cartilage and bone tissue remains to be achieved. Therefore, developing a method to perfectly and permanently repair the damaged cartilage and bone tissue is of significant clinical interest for patients with cartilage lesions and bone defects.

Tissue engineering, which emerged in the early 1990s, has become one of the most commonly used approaches for cartilage and bone tissue reconstruction and regeneration.^19,20,21,22^ Generally, an engineered tissue is composed of a scaffold, cells, and necessary growth factors.^23,24^ To fully reconstruct the damaged cartilage and bone tissue, it is important to synthesize biocompatible and biodegradable scaffolds that mimic the native features of the specific tissue, successfully transport cells and growth factors to the damaged tissue, and provide support to the newly formed tissue until it matures.^25^ Ideally, the scaffolds of both cartilage and bone tissue engineering should be porous, highly biocompatible, nontoxic, and capable of promoting cell differentiation and new tissue formation; they should also have stable mechanical properties, degrade in response to the formation of new tissue, facilitate the diffusion of nutrients and metabolites, adhere and integrate with the surrounding native tissue, and properly fill the injured site.^3^^24,26,27,28^

**Figure 1:**
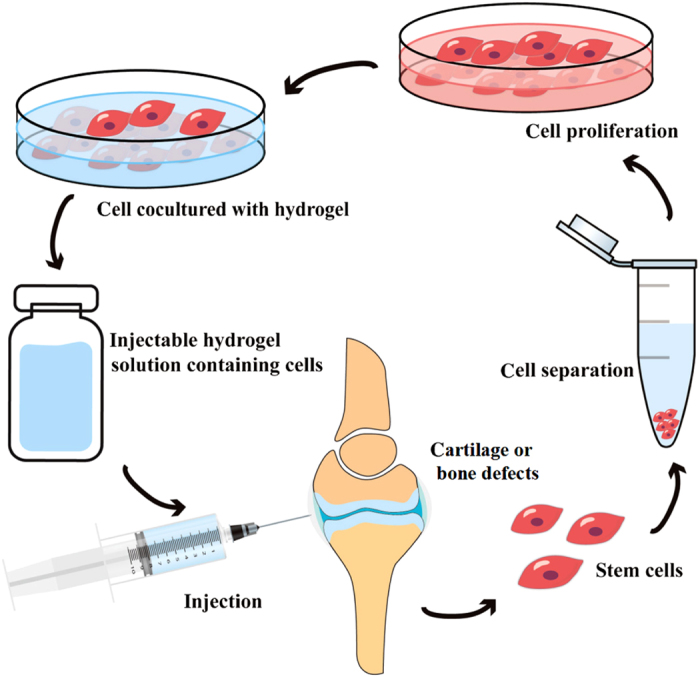
Schematic illustration of approaches to make injectable hydrogels for cartilage- and bone tissue-engineering applications.

Since the 1990s, a variety of biomaterials have been investigated and tested for cartilage- and bone tissue-engineering applications.^29,30,31,32,33,34,35,36,37,38^ Among all the biomaterials, hydrogels have received widespread interest, particularly for their use as scaffolds in cartilage and bone tissue engineering, owing to their structural similarity to the extracellular matrix (ECM) and their porous framework, which enables cell transplantation and proliferation.^39^ Hydrogels are three-dimensional (3D) cross-linked networks formed by hydrophilic homopolymers, copolymers, or macromers that swell in aqueous solution and provide an appropriate microenvironment similar to the ECM, thus facilitating the migration, adhesion, proliferation, and differentiation of chondrocytes and osteoprogenitor cells to osteoblasts, and efficiently delivering nutrients and growth factors.^39–42^ Recently, injectable hydrogels have attracted the attention of biomaterials scientists for cartilage- and bone tissue-engineering applications, because they can replace implantation surgery with a minimally invasive injection method and can form any desired shape, to match irregular defects.^3,43–47^ The schematic describing injectable hydrogels for cartilage- and bone tissue-engineering applications is illustrated in Figure 1.

Excellent biomaterials and appropriate fabrication methods play crucial roles in developing ideal injectable hydrogels that can function as scaffolds for cartilage- and bone tissue-engineering applications. A variety of biomaterials, both natural and synthetic, have been exploited to prepare injectable hydrogels; these biomaterials include chitosan,^43^ collagen or gelatin,^48,49^ alginate,^50^ hyaluronic acid,^51^ heparin,^52^ chondroitin sulfate,^53^ poly(ethylene glycol) (PEG),^54^ and poly(vinyl alcohol).^55^ Injectable hydrogels can be fabricated through both physical and chemical methods. Physically injectable hydrogels are spontaneously formed by weak secondary forces, whereas chemical hydrogels are usually formed by covalently cross-linking.^56–58^ On the basis of the concrete fabrication methods, injectable hydrogels can be classified as enzymatically cross-linked hydrogels,^59^ photo-cross-linked hydrogels,^60^ Schiff base cross-linked hydrogels,^61^ Michael addition-mediated hydrogels,^62^ click chemistry-mediated hydrogels,^44,63^ ion-sensitive hydrogels,^64^ pH-sensitive hydrogels,^65^ and temperature-sensitive hydrogels.^66,67^ Although injectable hydrogels prepared by different methods have been investigated for decades, there are scarcely any perfect injectable hydrogels that have been utilized in clinical regenerative medicine. Therefore, the development of an excellent injectable hydrogel for cartilage- and bone tissue-engineering applications is urgently needed. In this review, various biomaterials and fabrication methods for developing injectable hydrogels for cartilage- and bone tissue-engineering applications are discussed.

Even though many journal articles and reviews on injectable hydrogels for tissue engineering have been published, this is the first review that particularly focuses on both biomaterials and fabrication methods for developing novel injectable hydrogels, specifically for use in cartilage and bone tissue engineering. In this review, we provide a guide for selecting an appropriate biomaterial and fabrication method to prepare such injectable hydrogels. In addition, the biology of cartilage and the bony ECM is also discussed. Finally, perspectives on future injectable hydrogels for cartilage and bone tissue engineering are also discussed.

## THE BIOLOGY OF CARTILAGE AND THE BONY ECM

**Figure 2:**
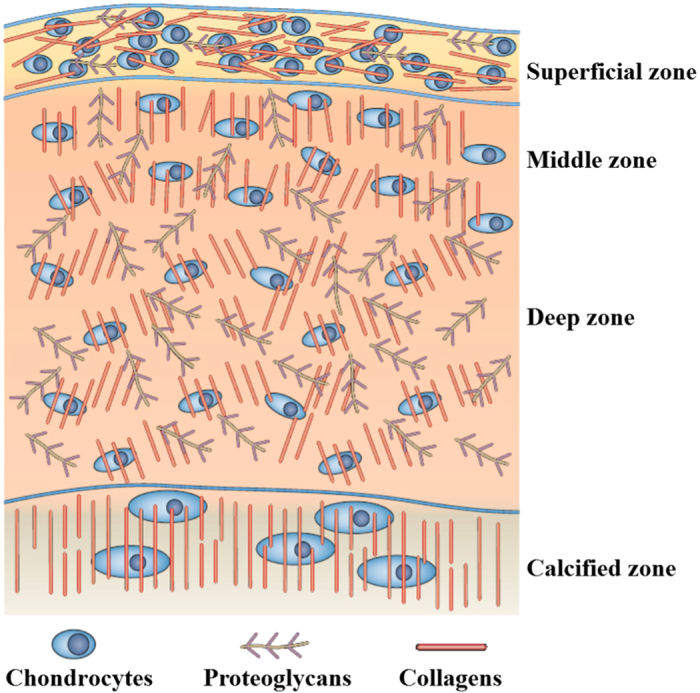
Schematic illustration of depth-dependent architecture of cartilage tissue. From the superficial zone to the deep zone, the proteoglycan content gradually increases. In the superficial zone, the collagen fibers are aligned parallel to the surface. Collagen fibers in the middle zone are unaligned and tangential to the cartilage surface. In the deep zone, collagen fibers are arranged radially. Finally, the collagen fibers in the calcified zone tend to arborize with little organization and mineralization.^72^

In cartilage and bone tissue engineering, detailed understanding of the biology of cartilage and the bony ECM is crucial in realizing successful cartilage and bone tissue regeneration. Cartilage is a fiber-reinforced composite material composed of chondrocytes surrounded by specialized ECM consisting of structural and functional proteins, glycoproteins, and glycosaminoglycans assembled in unique tissue-specific 3D microenvironment architectures.^68,69, 70,71^ The composition and structure of cartilage tissue are always depth-dependent (Figure 2) and can be divided into four different zones on the basis of collagen fiber alignment and proteoglycan composition.^71,72,73,74^ From the superficial zone to the deep zone, the proteoglycan content gradually increases. In the superficial zone, the collagen fibers are aligned parallel to the surface. Collagen fibers in the middle zone are unaligned and tangential to the cartilage surface. In the deep zone, collagen fibers are arranged radially. Finally, the collagen fibers in the calcified zone tend to arborize with little organization and mineralization.

**Figure 3:**
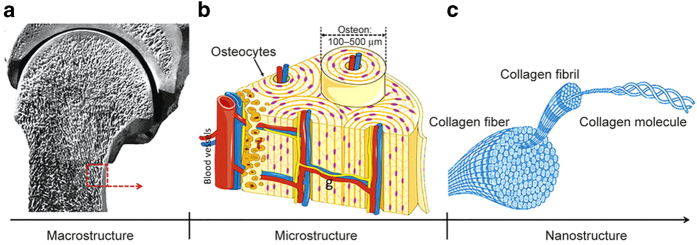
Schematic illustration of a distinct hierarchical structure of bone tissue. (**a**)At the macrostructural level, bone is composed of cortical bone and cancellous bone. (**b**) At the microstructural level, the cortical bone is made up of repeated units of osteon, which is characterized by 20–30 concentric layers of collagen fibers, called lamellae. The lamellae surround the central canal and contain various blood vessels and nerves. (**c**) At the nanostructural level, there are large numbers of collagen fibers, which are composed of periodic collagen fibrils and gaps between the collagen molecules. The calcium phosphate crystals and non-collagenous organic proteins are embedded in these gaps between collagen molecules.^76^

In contrast to cartilage tissue, bone is a highly vascularized biomineralized connective tissue with high mechanical strength and structural complexity.^57,75^ Natural bone tissue has a distinct hierarchical structural organization at the macrostructural, microstructural, and nanostructural levels (Figure 3).^76,77^ At the macrostructure level, bone can be distinguished into cortical bone and cancellous bone. At the microstructure level, the cortical bone is made up of repeated units of osteon, whereas the cancellous bone is composed of an interconnecting framework of trabeculae complemented with bone marrow-filled free spaces. Each osteon has 20–30 concentric layers of collagen fibers, called lamellae, which surround the central canal and contain various blood vessels and nerves. Finally, at the nanostructure level, there are large amounts of collagen fibers, calcium phosphate crystals, and non-collagenous organic proteins, which are the main components of the trabeculae and osteon units.^76^ The mechanical properties of bone tissue strongly depend on the specific structure and organization of the bony ECM.

This highly organized and complicated structure of the cartilage and bone is essential to support its biological functions. The composition of both cartilage and the bony ECM is highly complex. Normally, the native cartilage ECM is composed primarily of water, type II collagen, proteoglycans, hyaluronic acid, glycosaminoglycans, and elastin.^73,76, 78,79,80^ Unlike cartilage ECM, the bony ECM is composed of oriented collagen I fibers and nanocrystals of carbonated hydroxyapatite, and is complemented with a number of proteoglycans, glycoproteins, and sialoproteins.^81,82^ All components of both cartilage and the bony ECM, which are continuously synthesized, secreted, oriented, and modified by the chondrocytes or osteoblasts that they support, are essential for chondrocyte and osteoblast growth, development, maintenance, and regulate the biological activities of the native cartilage and bone tissue.^57,83,84^ Under physiological conditions, the ECM exists in a state of dynamic reciprocity with chondrocytes and osteoblasts, and provides a mechanical framework for supporting the cells.^70^ In addition, the ECM and ECM-incorporated growth factors, together with cytokines, provide a number of functional cues that affect chondrocyte and osteoblast metabolism, and secretion. Moreover, the microenvironment provided by the ECM is dynamic and regulated by factors, such as mechanical properties, pH, oxygen concentration, and hormonal actions, that affect tissue homeostasis and possible aberrations thereof.^69,85,86^ Eventually, the ECM not only regulates cell adhesion, migration, growth, differentiation, and apoptosis but also takes part in cytokine activity and intracellular signaling.^84,86^ The complexity of the ECM is essential for specific function of the cartilage and bone tissue, and plays an important role in keeping the physiological stability of the microenvironment. Thus, design and synthesis of novel biomaterials that imitate the natural ECM are of great significance in cartilage and bone tissue engineering, and regenerative medicine.

## INJECTABLE HYDROGELS PREPARED WITH DIFFERENT BIOMATERIALS

Various biomaterials have been exploited for the fabrication of injectable hydrogel scaffolds for cartilage tissue-engineering applications, including natural biomaterials and synthetic biomaterials.

### Natural biomaterial-based injectable hydrogels

Natural biomaterials have been widely investigated because of their perfect biocompatibility, biodegradability, and similarity to the ECM. Natural biomaterials recently investigated for use as injectable hydrogel preparations include chitosan, collagen/gelatin, alginate, fibrin, elastin, heparin, chondroitin sulfate, and hyaluronic acid.^3,46,50,52,53, 87,88,89,90,91^

#### Chitosan-based injectable hydrogels

Chitosan is a linear polysaccharide that is derived from natural chitin, which is composed of glucosamine and *N*-acetylglucosamine.^92,93,94, 95^ Recently, chitosan has become increasingly attractive as an injectable hydrogel for cartilage repair, owing to its structural similarity to cartilage glycosaminoglycan.^43,93,96^ Chen *et al*^48^ have fabricated a tough chitosan–gelatin hydrogel via an *in situ* precipitation method. This *in situ* formed hydrogel exhibits improved mechanical properties, and is biodegradable and biocompatible. Naderi-Meshkin*et al*^96^ have developed a chitosan-based injectable hydrogel via the combination of chitosan, glycerol phosphate, and the cross-linking agent hydroxyethyl cellulose. Systematic investigations of the viability, proliferation, and differentiation capacity of encapsulated mesenchymal stem cells in the hydrogel have indicated that this chitosan-based injectable hydrogel has a high potential for cartilage tissue engineering. To make stimuli-responsive injectable hydrogels, chitosan is usually combined with various chemical components. By combining chitosan–glycerophosphate with different concentrations of starch, Sá-Lima *et al*^97^ have successfully prepared a novel thermoresponsive chitosan–starch hydrogel that can be used as an injectable vehicle for cell delivery. Furthermore, Moreira *et al*^98^ have reported a bioactive thermogelling chitosan-based injectable hydrogel synthesized by combining chitosan, collagen, and bioactive glass nanoparticles. Chitosan is insoluble in water, but it can be dissolved in acetic acid solution. Therefore, chitosan-based hydrogels are obtained from chitosan–acetic acid solution, which requires tedious washing steps.^99^ To overcome such shortcomings, water-soluble chitosan derivatives have been introduced. For example, Kamoun^100^ has prepared a new class of nontoxic, injectable, biodegradable materials called *N*-succinyl chitosan-dialdehyde starch hybrid hydrogels. These hydrogels have shorter gelation times, limited water uptake, little weight loss, and considerably tighter hydrogel structures, thus making them preferable scaffolds for cartilage tissue engineering.

#### Collagen/gelatin-based injectable hydrogels

Collagen is the most abundant mammalian protein in the skin, connective tissue, ligaments, bone, and cartilage of the body.^101,102,103,104^ There are at least 19 types of collagen, such as type I, type II, type III, and type V.^101^ Recently, naturally derived collagen has been widely used to construct collagen-based scaffolds for various biomedical applications, particularly tissue engineering, because it has the favorable property of being weakly antigenic.^8,49,105^ Yuan *et al*^105^ have combined type I and type II collagens to construct a favorable injectable hydrogel whose compressive modulus can be regulated by changing the type I collagen content in the hydrogel. The chondrocytes embedded in the hydrogel maintain their natural morphology and secrete cartilage-specific ECM. Funayama*et al*^106^ have developed an injectable type II collagen hydrogel scaffold and have embedded chondrocytes in the collagen-based hydrogel and injected it into the damaged rabbit cartilage without a periosteal graft. At 8 weeks after the injection, favorable hyaline cartilage regeneration with good chondrocyte morphology was observed, and significant differences between the transplanted and control groups were observed after 24 weeks. Furthermore, collagen-based injectable hydrogels can be prepared by integrating collagen with other biomaterials. For example, Kontturi*et al*^107^ have developed an injectable, *in situ* forming type II collagen/hyaluronic acid hydrogel for cartilage tissue engineering. After encapsulation of chondrocytes and chondrogenic growth factor transforming growth factor-β_1_ into the hydrogel, the cell viability and proliferation, morphology, glycosaminoglycan production, and gene expression have been investigated. This hydrogel is able to maintain chondrocyte viability and characteristics, and it maybe a potential injectable scaffold for cartilage tissue engineering.

Gelatin is a natural protein derived from the degradation of collagen with high biocompatibility and biodegradability in physiological environments.^108,109^ Recently, use of gelatin to prepare injectable hydrogels has received popularity. Oh *et al*^110^ have designed and synthesized an interconnected, double thermoresponsive macroporous gelatin-based injectable hydrogel by stabilizing oil-in-water high internal phase emulsions, with gelatin-graft-poly(*N*-isopropyl acrylamide). In this injectable hydrogel, gelatin was chosen as the backbone of the amphiphilic graft copolymer to form high internal phase emulsions. The double thermoresponsive properties of the hydrogel promote proliferation and penetrate fibroblasts during cell seeding. Geng *et al*^111^ have prepared a gelatin-based injectable hydrogel from oxidized dextran, amino gelatin, and 4-arm PEG-acrylate through a two-step process. The attachment and spreading of preosteoblasts, as well as the encapsulated cell spreading and proliferation within the hydrogel indicate that the injectable hydrogel possesses favorable mechanical properties, biodegradability, and biocompatibility.

#### Hyaluronic acid-based injectable hydrogels

Hyaluronic acid, which interacts with chondrocytes through surface receptors such as CD44 and RHAMM,^112,113,114^ is a linear polysaccharide in the adult cartilage ECM and is composed of disaccharide units of glucuronic acid and *N*-acetylglucosamine.^115,116, 117^ Hyaluronic acid plays very important roles in cartilage and limb bud formation, mesenchymal cell condensation, chondrocyte matrix deposition, and chondrogenic differentiation.^73,118,119^ Therefore, hyaluronic acid is regarded as an ideal biomaterial for cartilage tissue repair. Yu *et al*^120^ have fabricated an injectable hyaluronic acid/PEG hydrogel with excellent mechanical properties for cartilage tissue engineering. Cells encapsulated in the hydrogel *in situ* demonstrate high metabolic viability and proliferation. In addition, taking advantage of its biocompatibility, structural similarity to glycosaminoglycan, and ready formation of ionic complexes of chitosan, Park *et al*^121^ have successfully fabricated an injectable chitosan–hyaluronic acid hydrogel utilizing hyaluronic acid and methacrylated glycol chitosan. Chondrocytes encapsulated in the hydrogel show excellent proliferation and increased deposition of cartilaginous ECM; considering these results, this hydrogel has great potential for cartilage tissue repair.

To overcome its poor mechanical properties, fast degradation, and hydrolytic reactions, hyaluronic acid is usually modified or combined with other biomaterials for practical applications.^113,122^ Palumbo *et al*^123^ have designed an *in situ* forming hydrogel by the addition of divinyl sulfone-functionalized inulin to two types of amino-functionalized hyaluronic acid derivatives, specifically pendant ethylenediamino and amino/octadecyl hyaluronic acids. The properties of the hydrogel indicate that the presence of pendant C18 chains improves the mechanical performances of hyaluronic acid-based hydrogels and decreases their susceptibility to hyaluronidase hydrolysis. Furthermore, encapsulated bovine chondrocytes in the hydrogel result in high viability and proliferation. Domingue *et al*^124^ have used cellulose nanocrystals as nanofillers to develop a new class of reinforced hyaluronic acid-based injectable hydrogels, which comprise adipic aciddihydrazide-modified hyaluronic acid and aldehyde-modified hyaluronic acid reinforced by the aldehyde-modified cellulose nanocrystals. The biological performance of the developed hydrogel has been evaluated on the basis of the incorporation of human adipose-derived stem cells. The hydrogel has been found to possess preeminent cell-supportive properties and to spread well within the volume of gels, in addition to exhibiting pronounced proliferative activity.

#### Fibrin-based injectable hydrogels

Fibrin, which is regarded as a favorable cell-transplantation matrix that can enhance cell attachment, proliferation, differentiation, and migration in a 3D scaffold, is a natural fibrous protein involved in blood clotting.^125,126,127^ In previous studies, fibrin, alone or in combination with other materials, has been used to synthesize scaffolds for cartilage tissue-engineering applications.^128,129,130,131^ Benavides *et al*^132^ have applied fibrin-based hydrogels, together with PEG and human amniotic fluid-derived stem cells, to develop a novel injectable hydrogel system that is able to induce a fibrin-driven angiogenic host response and promote *in situ* amniotic fluid-derived stem cell-derived neovascularization. Almeida *et al*^133^ have developed an injectable, cartilaginous ECM microparticle-functionalized fibrin-based hydrogel, which transforms growth factor transforming growth factor-β_3_ into a putative therapeutic for articular cartilage regeneration. The capacity of the hydrogel to promote chondrogenes is of freshly isolated stromal cells *in vivo* suggests that the hydrogel can induce cartilage formation and has the potential for cartilage repair, and thus may have the potential to overcome several current challenges related to cartilage tissue engineering. In addition, because alginate microbeads are stable and biocompatible, this hydrogel has been widely applied among injectable hydrogel systems for tissue regeneration.^125^ Hwang *et al*^134^ have developed a novel hybrid hydrogel system using alginate particles and a fibrin matrix. In this hydrogel, the introduction of alginate particles into a fibrin matrix enhances cellular mobility and proliferation, volume retention, and vascularization *in vivo*, thus making the injectable hybrid system a desirable approach for cartilage tissue-engineering applications.

#### Alginate-based injectable hydrogels

Alginate, which consists of guluronic and mannuronic acids, is a polysaccharide extracted from brown algae (Phaeophyceae).^50,135,136^ Alginate has become one of the most commonly used biomaterials in injectable hydrogel preparation for cartilage tissue-engineering applications, owing to its favorable scaffold forming, non-immunogenicity, and non-toxicity.^135, 137,138,139^ For example, Balakrishnan *et al*^140^ have produced a rapidly gelling, oxidized alginate-based injectable hydrogel by self-cross-linking periodate-oxidized alginate and gelatin in the presence of borax. The hydrogel integrates well with the cartilage tissue in addition to exhibiting negligible inflammatory and oxidative stress responses. Moreover, chondrocytes encapsulated in the hydrogel have favorable viability, and exhibit a normal phenotype in terms of proliferation and migration within the matrix, thus suggesting that the hydrogel is a promising injectable, cell-attracting adhesive scaffold for cartilage tissue engineering.

However, there is a drawback to using an injectable alginate hydrogel: it is not strong enough to maintain the structural shape of the regenerated tissue.^141^ Therefore, alginate is usually modified or used in combination with other biomaterials to improve its mechanical properties. Zhao *et al*^142^ have devised a fully injectable and mechanically strong calcium phosphate–alginate cement hydrogel system. The mechanical properties of the hydrogel are much better than those of previous injectable polymeric and hydrogel carriers, and the encapsulated cells are viable, exhibit osteodifferentiation, and secrete bone minerals. Furthermore, owing to its lack of cell adhesion ability, alginate is usually blended with other polymers.^143,144^ An injectable, biodegradable, oxidized alginate/hyaluronic acid hydrogel has been prepared by Park and Lee.^143^ At 6 weeks after injection of the hydrogel with primary chondrocytes into mice, effective cartilage regeneration has been observed. In another study, a class of biocompatible and biodegradable alginate-based hydrogel blend has been synthesized by using alginate and *O*-carboxymethyl chitosan with the addition of fibrin nanoparticles.^144^ Evaluation of the swelling ratio, degradation profile, compressive strength, and elastic module have indicated that alginate/*O*-carboxymethyl chitosan forms a preferable blend for tissue-engineering applications.

#### Heparin-based injectable hydrogels

Heparin, which is best known for its anticoagulant properties, is a negatively charged, highly sulfated, linear polysaccharide composed of repeating disaccharide units of 1,4-linked uronic acid and glucosamine residues.^145,146,147,148^ Owing to its negatively charged functional groups, heparin can interact with proteins, including ECM proteins, growth factors, and chemokines, which plays important roles in many biological processes, such as triggering multiple downstream signaling pathways and controlling cellular proliferation, and differentiation.^149,150,151,152,153,154^ As a result, heparin has widely been used for the fabrication of injectable hydrogels that control the delivery of growth factors in tissues, especially during cartilage tissue repair.^153, 155,156,157, 158^ For example, Jin *et al*^159^ have used horseradish peroxidase (HRP)-mediated co-cross-linking to form dextran–tyramine (Dex–TA) and heparin–tyramine injectable hydrogel conjugates whose swelling and mechanical properties can be controlled for cartilage tissue-engineering applications. Chondrocytes incorporated in the hydrogel exhibit favorable viability and proliferation, with increased production of chondroitin sulfate and abundant collagen content. In addition, heparin-based injectable hydrogels can also be combined with other scaffolds to reinforce its curative effects. Such a strategy has been attempted by Kim *et al*,^160^ who have combined the advantages of a porous gelatin-incorporated poly(L-lactide-*co*-*ε*-caprolactone) scaffold and heparin-based injectable hydrogels to produce a scaffold/hydrogel composite for delivering chondrocytes to repair partial thickness cartilage defects. Cells encapsulated in the scaffold/hydrogel composite exhibit enhanced expression of chondrogenic genes and increased the production of glycosaminoglycans. In addition, significant cartilage formation that integrates well with the surrounding natural cartilage tissue has been observed when this composite has been used to repair partial thickness defects of rabbit knees. All of these results indicate that the scaffold/hydrogel composite is a promising scaffold system for cartilage regeneration.

#### Elastin-based injectable hydrogels

Elastin is an insoluble, polymeric, elastic protein found in soft tissue, such as skin, blood vessels, and lungs.^161,162^ Currently, elastin-based biomaterials are widely used in tissue engineering, especially in fabricating injectable hydrogels for cartilage tissue engineering, because elastin not only improves local elasticity but also facilitates cellular interactions and signaling during neoplastic tissue formation.^162,163^ For instance, Fathi *et al*^87^ have fabricated a highly cytocompatible and injectable elastin-based hydrogel with alterable gelation characteristics, favorable mechanical properties, and good structural stability. This hydrogel is generated by the synthesis of a polymer (PNPHO) by functionalizing poly(*N*-isopropylacrylamide-*co*-polylactide-2-hydroxyethylmethacrylate-*co*-oligo(ethylene glycol)monomethyl ether methacrylate with succinimide ester groups, then covalently attaching elastin to PNPHO via interaction of its primary amine groups with the ester groups of PNPHO in aqueous solution. The elastin-*co*-PNPHO solutions are injectable and convert into hydrogels *in situ* at 37 °C without any cross-linking reagent. In addition, this elastin-based injectable hydrogel shows favorable structural stability and mechanical properties as well as preferable cyto-biocompatibility, thus making it a favorable candidate for cartilage tissue-engineering applications.

#### Chondroitin sulfate-based injectable hydrogels

Chondroitin sulfate, which is composed of sulfated disaccharide repeating units with 1–3 linkages of D-glucuronic acid and *N*-acetylgalactosamine, is an abundant anionic linear polysaccharide present in connective tissue and bones, and is an important component of cartilage in the body.^164,165,166,167^ Chondroitin sulfate plays important roles in many biological processes such as intracellular signaling, cell recognition, the connection between ECM components and cell-surface glycoproteins, and chondrocyte phenotype regulation, as has widely been investigated in cartilage tissue engineering.^168,169,170,171^ Wiltsey *et al*^172^ have developed a poly(*N*-isopropylacrylamide)-graft-chondroitin sulfate-based injectable hydrogel scaffold, which acts as a favorable adhesive interface with surrounding tissue. The hydrogel system has been demonstrated to have improved mechanical properties at 37 °C, enhanced adhesive tensile strength (ranging from 0.4 to 1 kPa), and no cytotoxicity to human embryonic kidney 293 cells. Chen *et al*^173^ have successfully developed a novel injectable pullulan/chondroitin sulfate composite hydrogel, synthesized under physiological conditions, for cartilage tissue engineering. The hydrogel system is very cytocompatible, enhances cell proliferation, and increases cartilaginous ECM deposition, thus showing promise for cartilage tissue repair.

### Synthetic biomaterial-based injectable hydrogels

Compared with natural biomaterials, synthetic biomaterials, owing to their enhanced controllability and reproducibility, enable the systematic study of cell–matrix interactions.^57^ To date, several degradable synthetic polymers have been studied for the development of injectable hydrogels for cartilage tissue engineering; these polymers include PEG,^114, 174,175,176,177^ poly(L-glutamic acid),^178,179^ poly(vinyl alcohol),^180^ poly(propylene fumarate),^181^ α,β-poly(*N*-hydroxyethyl)-DL-aspartamide,^182^ PEG-poly(*N*-isopropyl acrylamide) (PNIPAAm),^183^ methoxy polyethylene glycol,^184^ and methoxy polyethylene glycol–poly(*ε*-caprolactone).^185^ For example, Yan *et al*^186^ have reported a novel poly(L-glutamic acid)-based injectable hydrogel. Preliminary studies of the hydrogel have demonstrated successful injectability, rapid *in vivo* gelling, excellent cell growth, satisfactory mechanical stability, and favorable ectopic cartilage formation. Skaalure*et al*^187^ have developed a new cartilage-specific, degradable hydrogel based on PEG and have encapsulated bovine chondrocytes from different sources in the hydrogel for cartilage tissue engineering. This new PEG-based injectable hydrogel shows promise for cartilage regeneration. Moreover, De France *et al*^188^ have designed an *in situ* gelling nanocomposite hydrogel based on poly(oligoethylene glycol methacrylate) and rigid rod-like cellulose nanocrystals. This injectable hydrogel possesses enhanced mechanical properties, increased stability and gelation rates, and decreased swelling ratios.

However, synthetic biomaterials are not very biocompatible, and, as compared with natural biomaterials, they lack biological activity. The most common strategy used to solve this problem is modifying or combining synthetic biomaterials with bioactive polymers. For example, Yan *et al*^178^ have fabricated a series of injectable poly(L-glutamic acid)/alginate (PLGA/ALG) hydrogels by self-cross-linking hydrazide-modified poly(L-glutamic acid) and aldehyde-modified alginate. This injectable PLGA/ALG hydrogel exhibits attractive properties for future application in cartilage tissue engineering. In addition, Yu *et al*^120,189^ have fabricated two hyaluronic acid/PEG-based injectable hydrogels. Both hydrogels possess good mechanical properties and short gelation times, and the cells encapsulated in the hydrogels exhibit high metabolic viability and proliferation, thus indicating that both hydrogels have great potential in cartilage tissue engineering.

## INJECTABLE HYDROGELS FABRICATED VIA DIFFERENT APPROACHES

There are various approaches available for the fabrication of injectable hydrogels; depending on the approach used, injectable hydrogels can be divided into physical hydrogels and chemical hydrogels. Physical hydrogels are spontaneously formed by weak secondary forces, which respond to the changes in temperature, pH, or ionic concentration.^63,190,191^ Chemical hydrogels are produced through a variety of chemical processes, for example, enzymatic cross-linking, Schiff base cross-linking, Michael additions, click chemistry, and photo-cross-linking.^44,56,62,63,192,193^

### Injectable hydrogels by physical methods

#### Temperature-sensitive injectable hydrogels

Injectable hydrogels that are sensitive to temperature changes have recently attracted substantial attention for applications in cartilage tissue engineering, because of their gelation ability at physiological temperature. These injectable hydrogels are present in aqueous form at room temperature, but they rapidly gel at physiological temperature before solidifying in the desired tissue.^194,195^ The threshold temperature at which hydrogels transform from a solution to a hydrogel state is defined as the lower critical solution temperature. The most useful characteristic of temperature-sensitive hydrogels is that they can undergo a phase transition without any chemical stimulus. To date, the most common explanation of the phase transition mechanism of temperature-sensitive injectable hydrogels is that when the temperature changes, there is a change in the hydration state favoring intra- and inter-molecular hydrogen bonding, thus eventually changing the hydrogel solubility.^196,197^ Therefore, to make injectable hydrogels that are sensitive to temperatures, temperature-sensitive polymers such as poly(lactic-*co*-glycolic acid)–PEG,^194^ poly(*N*,*N*-diethylacrylamide),^195^ PNIPAAm,^197^ and poly(ethylene glycol-b-[DL-lactic acid-*co*-glycolic acid]-b-ethylene glycol)^198^ are needed.

PNIPAAm, an inverse temperature-sensitive polymer derived from polyacrylic acid, has become one of the most commonly used temperature-sensitive polymers, owing to its rapid phase transition at its ~32 °C lower critical solution temperature.^199,200,201^ However, linear PNIPAAm is not stable at physiological temperature, thus requiring the modification of other polymers to improve the stability and mechanical properties. Klouda*et al*^202^ have studied the effects of the macromer end group, acrylate or methacrylate, and the effects of fabrication conditions on the degradative and swelling properties of PNIPAAm-based injectable hydrogels. When immersed in cell culture medium at physiological temperature, the hydrogels maintain constant swelling, and exhibit no observable degradation over 8 weeks; the methacrylated hydrogels show greater swelling than their acrylated analogs. Another temperature-sensitive PNIPAAm-based injectable hydrogel, synthesized by functionalizing PNIPAAm with methacrylate groups by degradable phosphate ester bonds, has transition temperatures between room temperature and physiological temperature.^203^ Making temperature-sensitive injectable hydrogels by modifying PNIPAAm with natural polymers is another strategy to optimize their stability and mechanical properties. Ren *et al*^204^ have grafted temperature-sensitive PNIPAAm onto gelatin via atom transfer radical polymerization, creating a hydrogel that successfully undergoes a sol-to-gel transition at physiological temperature. Tan *et al*^205^ have synthesized a temperature-sensitive injectable hydrogel whose lower critical solution temperature is ~35 °C, by grafting PNIPAAm-COOH with a single carboxy end group onto aminated alginate through amide bond linkages. In addition, the hydrogel is not cytotoxic and preserves the viability of the entrapped cells, thus making it suitable as a cell delivery vehicle for cartilage tissue-engineering applications.

#### pH-sensitive injectable hydrogels

Injectable hydrogels sensitive to pH value show significant potential in regenerative medicine. To obtain pH-sensitive injectable hydrogels, it is necessary to incorporate the hydrogel with a pH-sensitive moiety such as the polyelectrolyte *N*-palmitoylchitosan,^65^ polyacrylic acid,^206^ oligomeric sulfamethazine,^207^ and sulfamethazine oligomers (SMOs).^208^ For example, Shim *et al*^209^ and Kim *et al*^191^ have synthesized a pH-sensitive injectable hydrogel by adding pH-sensitive SMOs to both ends of a temperature-sensitive poly(*ε*-caprolactone-*co*-lactide)–PEG–poly(*ε*-caprolactone-*co*-lactide) (PCLA–PEG–PCLA) block copolymer. This pH-sensitive SMO–PCLA–PEG–PCLA–SMO injectable hydrogel exists in solution at high pH (pH 8.0), but rapidly changes into a stable gel under physiological conditions (pH 7.4). Kim *et al*^191^ have encapsulated human mesenchymal stem cells and recombinant human bone morphogenetic protein-2 into the hydrogels under physiological conditions and injected the mixture into the backs of mice. Histological studies observing human mesenchymal stem cell differentiation for 7 weeks have revealed mineralized tissue formation and high levels of alkaline phosphatase activity in the mineralized tissue.

#### Other physical injectable hydrogels

Other physical injectable hydrogels, such as ion-sensitive and stress-sensitive hydrogels, for cartilage tissue-engineering applications have also been reported.^62,63,64^ For instance, Park *et al*^64^ have prepared an ionically cross-linkable hyaluronate-grafted-alginate hydrogel that easily forms gels in the presence of calcium ions and has been demonstrated to be useful in cartilage regeneration by the subcutaneous injection of primary chondrocyte-encapsulated hyaluronate-grafted-alginate into the dorsal region in a mouse model. Except for the novel methods of developing physical injectable hydrogels, determining how to improve the biocompatibility, biodegradability, mechanical properties, and the *in vivo* maintenance of structural integrity of correlated biomaterials are further research topics for the design of physical injectable hydrogels.

### Injectable hydrogels by chemical methods

#### Injectable hydrogels by enzymatic cross-linking

Recently, the use of the enzymatic cross-linking method applied to the development of novel injectable hydrogels has drawn attention, owing to the fast gelation, high site specificity, ability to work at normal physiological conditions, and low cytotoxicity.^210–215^ Several enzyme-mediated cross-linking systems have been applied to synthesizing injectable hydrogels for cartilage tissue-engineering applications, including transglutaminase, tyrosinase, phosphopantetheinyl transferase, lysyl oxidase, plasma amine oxidase, phosphatase, thermolysin, β-lactamase, and peroxidase.^215^ Among them, HRP is the most commonly used enzyme in synthesizing injectable hydrogels. HRP is a single-chain β-type hemoprotein that catalyzes the conjugation of phenol and aniline derivatives in the presence of H_2_O_2_.^215,216^ The HRP-mediated cross-linking system covalently binds the phenol-conjugated polymers to the ECM proteins of the surrounding native tissue and thus is beneficial in maintaining the structural integrity of the wound tissue.^217^

**Figure 4:**
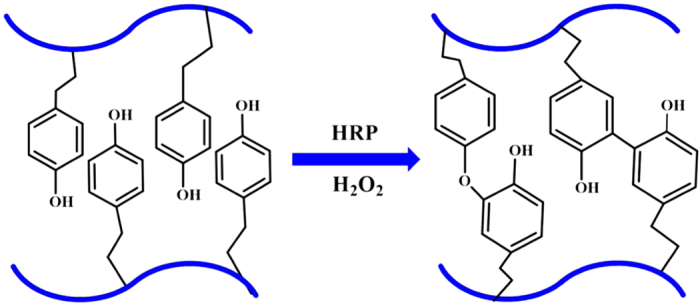
Schematic illustration of injectable hydrogels prepared by the enzymatic cross-linking method with horseradish peroxidase (HRP) and H_2_O_2_.

Both natural and synthetic polymers that contain phenol groups or are functionalized with tyramine, tyrosine, or other aminophenol molecules can be enzymatically cross-linked by HRP (Figure 4).^218,219,220^ For example, Wang *et al*^221^ have reported an HRP-mediated gelatin–hydroxyphenylpropionic acid-based injectable hydrogel for ectopic cartilage formation and early-stage osteochondral defect repair. The reported hydrogel was fabricated by oxidative coupling of hydroxyphenylpropionic acid moieties, catalyzed by HRP and H_2_O_2_. Jin *et al*^222^ have also enzymatically cross-linked Dex–TA conjugates in the presence of HRP and H_2_O_2_ to prepare an injectable hydrogel for cartilage tissue repair. Chondrocytes encapsulated in the Dex–TA hydrogels have been found to retain their viability and normal morphology after 2 weeks, and to secrete glycosaminoglycans and collagen type II after culturing for 14 and 21 days, thus indicating that the enzymatically cross-linked injectable Dex–TA hydrogels are promising for cartilage tissue-engineering applications.

#### Injectable hydrogels by Schiff base cross-linking

**Figure 5:**
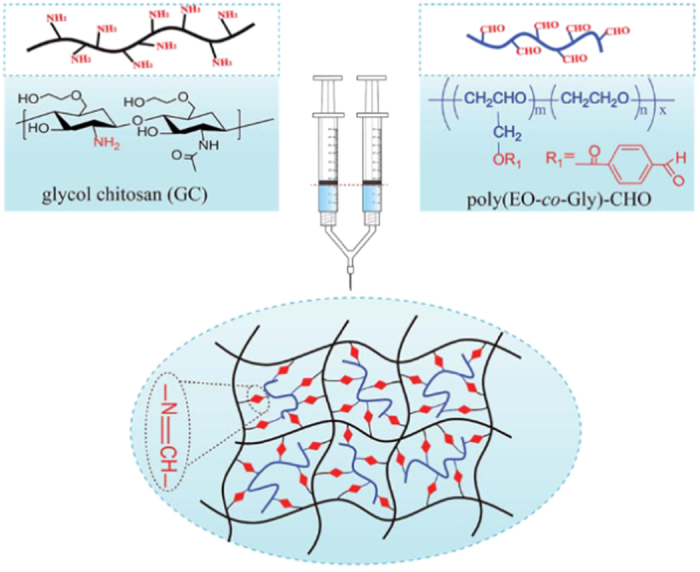
Schematic illustration of injectable hydrogels prepared by Schiff base cross-linking between aqueous solutions of GC and poly(EO-*co*-Gly)-CHO.^230^

Schiff base reactions have been widely used for synthesizing injectable hydrogels for cartilage regeneration applications, owing to the mild reaction conditions and high reaction rate, as well as the ability to form imine bonds between amino and aldehyde groups without any external stimuli or additional reagents under physiological conditions.^92, 223,224,225,226,227,228^ Chitosan is an excellent biomaterial for preparing injectable hydrogels via Schiff base cross-linking, owing to the abundant amino groups on its backbone. For example, Cheng *et al*^229^ have reported an injectable chitosan-based polysaccharide hydrogel for cell and protein delivery, which is cross-linked via an imine bond resulting from the Schiff base reaction between the amino functionalities of chitosan and the aldehyde groups of dextran aldehyde in aqueous solutions. Cao *et al*^230^ have utilized a multi-benzaldehyde-functionalized PEG analog, poly(ethylene oxide-*co*-glycidol)-CHO(poly(EO-*co*-Gly)-CHO), and glycol chitosan to successfully develop an injectable hydrogel system for cartilage tissue repair, which was chemically cross-linked through a Schiff base reaction between amino groups of glycol chitosan and aldehyde groups of poly(EO-*co*-Gly)-CHO under physiological conditions *in situ* (Figure 5). In addition, other biomaterial-based injectable hydrogels coupled by Schiff base cross-linking have been widely investigated. Most recently, Ma *et al*^231^ have developed a biodegradable and injectable polymer–liposome hydrogel by using aldehyde-modified xanthan gum and phosphatidylethanolamine liposomes, which are chemically cross-linked by a Schiff base reaction between the aldehyde groups of aldehyde-modified xanthan gum and amino groups of PE liposomes. This xanthan gum-based liposome hydrogel has many advantages, such as rapid preparation at room temperature, ready biodegradation by enzymes, excellent self-healing capability, and the ability to maintain favorable cell viability.

#### Injectable hydrogels by Michael addition

**Figure 6:**
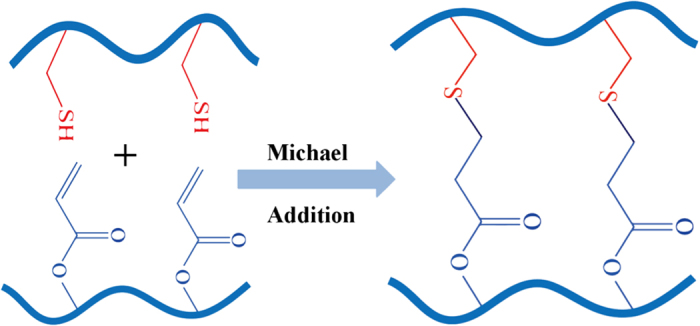
Schematic illustration of injectable hydrogels prepared by the Michael addition cross-linking method.

The Michael addition reaction, which is the nucleophilic addition of a carbanion or a nucleophile to an α,β-unsaturated carbonyl compound (Figure 6), is another commonly used approach to prepare injectable hydrogels, owing to its reaction under physiological conditions and controllable reaction time.^193, 232,233,234,235,236,237,238,239^ Hyaluronic acid, chitosan, and PEG are frequently used biomaterials for injectable hydrogel preparation via the Michael addition reaction for cartilage tissue engineering under physiological conditions.^114, 240,241,242^ For example, Calogero *et al*^243^ have prepared two kinds of hyaluronic acid-based injectable hydrogels by Michael addition, using the amino derivative of hyaluronic acid (HA-EDA), *α*-elastin-grafted HA-EDA, and α,β-poly(*N*-2-hydroxyethyl)-DL-aspartamidederivatized with divinylsulfone. The swelling and degradation profile as well as its ability to incorporate viable articular chondrocytes of the injectable hydrogel indicate that this injectable hydrogel scaffold possesses desired properties for the treatment of articular cartilage damage under physiological conditions.

#### Injectable hydrogels by click chemistry

**Figure 7:**
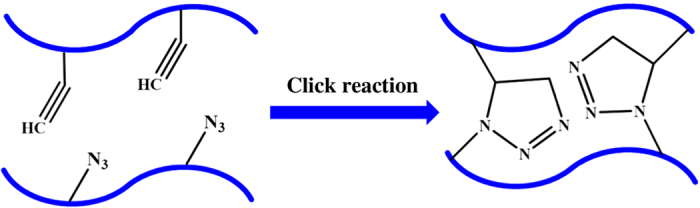
Schematic illustration of injectable hydrogels prepared by click chemistry.

Click chemistry refers to a synthetic concept involving a wide range of reactions (Figure 7), including copper-catalyzed azide-alkyne cyclo-addition reactions,^244,245,246^ Diels–Alder reactions,^120^ the thiol-ene reactions,^247,248^ tetrazine–norbornene chemistry,^249^ thiol-epoxy,^250^ and thiol-maleimide couplings.^251^ These reactions have shown great promise for the development of injectable hydrogels, owing to their rapid polymerization kinetics and low reactivity with cellular components.^252,253,254^ For example, Kaga *et al*^255^ have fabricated a dendron–polymer–dendron conjugate-based injectable hydrogel through radical thiol-ene “click” reactions. In this fabrication process, the dendron–polymer conjugates were prepared through an azide-alkyne “click” reaction of alkene-containing polyester dendrons, bearing an alkyne group at their focal point, with linear PEG-bisazides. The sequential thiol-ene “click” reaction uses a tetrathiol-based cross-linker to cross-link these alkene-functionalized dendron–polymer conjugates, thus resulting in clear and transparent hydrogels.

#### Injectable hydrogels by photo-cross-linking

**Figure 8:**
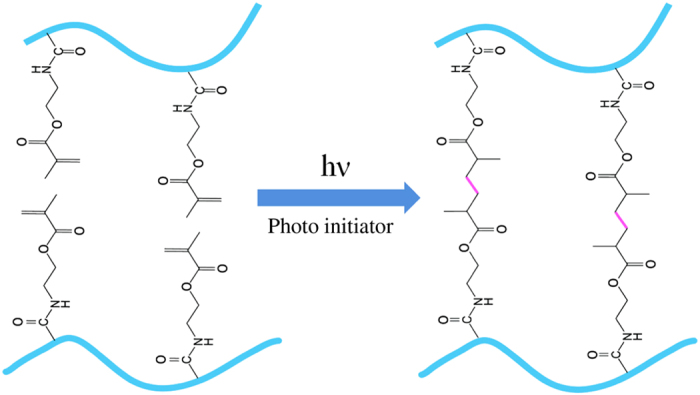
Schematic illustration of injectable hydrogels prepared by the photo-cross-linking method. Reprinted with permission from ref. 256 2009 Elsevier Publishing Group.

Photo-cross-linking is a complex process, consisting of initiation, propagation, and termination steps, triggered by electromagnetic radiation in the visible and ultraviolet regions (Figure 8).^256,257^ First, free radicals are created by the excitation of photoinitiators, as a result of the illumination in the initiation step. Then, long kinetic chains are cross-linked by propagating the radicals through unreacted double bonds in the propagation step, and this is followed by a termination step, which is characterized by the end of cross-linking in the 3D polymeric network.^257^ In recent years, photo-cross-linking methods have been widely applied to prepare injectable hydrogels for cartilage tissue engineering because of the ability to control the timing and location of cross-linking under physiological conditions.^258,259,260,261,262,263,264,265,266^ For example, Papadopoulos *et al*^267^ have developed a poly(ethylene glycol)dimethacrylate copolymer-based injectable hydrogel by photo-cross-linking for cartilage tissue-engineering applications. Swine auricular chondrocytes have been encapsulated into PEGDM copolymer hydrogels composed of degradable (PEG-4,5 LA-DM) and nondegradable PEGDM macromers in a 60:40 molar ratio. The histological, biochemical, and integrative features of the neocartilage indicate that the viability, proliferation, and normal secretion of glycosaminoglycan and hydroxyproline contents of the seeded chondrocytes are maintained, and the neocartilage resembles the native swine auricular cartilage, thus indicating the promise of these hydrogels in cartilage tissue-engineering applications.

## INJECTABLE HYDROGELS FOR BONE TISSUE ENGINEERING

Bone defects have become one of the leading causes of morbidity and disability among elderly people worldwide.^268,269^ Although autografting is regarded as the gold standard for bone defect repair, it is limited by the donor-site morbidity and uncertain adverse effects.^270^ Therefore, bone tissue engineering has attracted considerable attention from researchers as a promising strategy for repairing bone defects without the limitations and shortcomings of using either bone autografts, allografts, or xenografts.^271^

Recently, various injectable hydrogels with good moldability and 3D structures have been widely investigated for use in bone tissue engineering. Among the biomaterials used for preparing injectable hydrogels, alginate is one of the most investigated biomaterials used in bone tissue engineering.^135^ Matsuno *et al*^272^ have developed a novel injectable 3D hydrogel for bone tissue engineering that uses β-tricalcium phosphate beads and alginate as a scaffold. Mesenchymal stem cells 3D-cultured within the hydrogel have been implanted subcutaneously for *in vivo* experiments, and have indicated that the scaffold can favorably support osteogenic differentiation. Han *et al*^273^ have prepared an injectable calcium silicate/sodium alginate hybrid hydrogel by incorporating calcium silicate into an alginate solution. In 30 s to 10 min, this hydrogel undergoes internal *in situ* gelling when calcium ions are released from calcium silicate with the introduction of D-gluconic acid *δ*-lactone. Moreover, the hydrogel efficiently promotes the adhesion, proliferation, and differentiation of osteogenic and angiogenic cells. Chitosan is another commonly used biomaterial for synthesizing injectable hydrogels in bone tissue engineering.^274^ Dessi *et al*^275^ have successfully developed a thermosensitive chitosan-based hydrogel cross-linked with β-glycerophosphate and reinforced by physical interactions with β-tricalcium phosphate. The hydrogel simulates natural bone tissue and supports cellular activity and undergoes a sol–gel transition at physiological temperature with typical rheological properties. Meanwhile, owing to the properties of collagen, this hydrogel enhances cell adhesion and proliferation. Ding *et al*^276^ have incorporated collagen into the chitosan/β-glycerophosphate system to synthesize an injectable chitosan/β-glycerophosphate/collagen-based hydrogel scaffold for bone tissue engineering. Mesenchymal stem cells co-cultured in the hydrogel have been demonstrated to be capable of supporting neovascularization and osteogenic lineage differentiation. In recent years, synthetic biomaterials-based injectable hydrogels for bone tissue engineering have attracted attention. Jang *et al*^277^ have investigated an injectable *in vivo* forming hydrogel scaffold made of methoxy polyethylene glycol-b-polycaprolactone block copolymer for bone tissue engineering. Differentiated osteoblasts encapsulated in the hydrogel exhibit characteristic expression of osteonectin, osteopontin, and osteocalcin. Vo *et al*^278^ have designed an *N*-isopropylacrylamide/gelatin microparticle-composite hydrogel. The gelatin microparticles incorporated in the hydrogel enhance bony bridging and mineralization within the defect and direct bone-implant contact. After encapsulation of mesenchymal stem cells in the hydrogel, significant tissue infiltration and osteoid formation have been observed, thus suggesting that the hydrogel system facilitate bone ingrowth and integration.

To improve the mechanical properties and mineralization of the scaffold in bone tissue engineering, inorganic materials are usually introduced with hybrid hydrogels. Given that hydroxyapatite (HA) is one of the major inorganic components in bone tissue,^279^ Fu *et al*^280^ have prepared a novel three-component injectable thermosensitive hydrogel composite composed of triblock PEG–PCL–PEG copolymer, collagen, and nanohydroxyapatite. This hydrogel composite has a good interconnected porous structure in addition to excellent thermosensitivity. Furthermore, *in vivo* studies have demonstrated that the PECE/collagen/nanohydroxyapatite hydrogel has good biocompatibility and exhibits better performance in guided bone regeneration than in the self-healing process, thus indicating its great promise for bone tissue engineering. Furthermore, Jiao *et al*^281^ have synthesized an *in situ* cross-linkable citric acid-based biodegradable PEG maleate citrate/HA hydrogel. Huang *et al*^282^ have fabricated an injectable nanohydroxyapatite/glycol chitosan/hyaluronic acid composite hydrogel. MC-3T3-E1 cells incorporated in the hydrogel attach and spread well after 7 days of co-incubation, thus suggesting that the hydrogel’s potential application in bone tissue engineering. Lin *et al*^283^ have designed an injectable and thermosensitive hydrogel composite composed of poly(lactic acid-*co*-glycolic acid)-g-PEG and HA for its potential application in bone tissue engineering. The addition of HA into the hydrogel enhances the mechanical properties and bioactivity of the hydrogel. Most recently, an injectable alginate/HA hydrogel scaffold, combined with gelatin microspheres (GMs), has been reported by Yan *et al.*^284^ In this hydrogel, HA and GMs successfully improve the mechanical properties of the scaffold, thus demonstrating that the HA and GMs double-integrated alginate-based hydrogel has a suitable physical performance and bioactive properties. Thus, the hydrogel shows great potential for local treatment of pathologies involving bone defects. Moreover, taking advantage of the structural and regulatory cellular functions of zinc (Zn) and its ability to promote osteoblastogenesis and suppress osteoclastogenesis,^285^ Niranjan *et al*^286^ have reported a thermosensitive hydrogel, containing Zn, chitosan, and β-glycerophosphate, for bone tissue engineering. Furthermore, Dhivya*et al*^287^ have designed an injectable thermosensitive zinc-doped chitosan/nanohydroxyapatite/β-glycerophosphate-based hydrogel. *In vivo* studies in a ratbone-defect model system have indicated the potential of the hydrogel for accelerating bone formation at molecular and cellular levels. Other inorganic materials such as nanosilica and Bioglass have been studied for the preparation of hybrid hydrogel systems.^288,289^ For example, Vishnu Priya *et al*^290^ have developed an injectable hydrogel system by using chitin and poly(butylene succinate) loaded with fibrin nanoparticles and magnesium-doped Bioglass. This hydrogel system enhances the initiation of differentiation and expression of alkaline phosphatase and osteocalcin, thus indicating its promise for regenerating irregular bone defects.

## Conclusions and perspectives

Injectable hydrogels are promising scaffolds for cartilage and bone tissue engineering, owing to their minimal invasive properties and ability to match irregular defects. In this review, we summarized many novel injectable hydrogels prepared by a variety of biomaterial and fabrication techniques for cartilage- and bone tissue-engineering applications. First, injectable hydrogels fabricated from both natural biomaterials and synthetic biomaterials were reviewed. Natural biomaterials such as chitosan, collagen/gelatin, alginate, fibrin, elastin, heparin, and hyaluronic acid are among the most commonly used biomaterials for the preparation of injectable hydrogels, owing to their perfect cyto-biocompatibility, biodegradability, low cytotoxicity, and similarity to the natural cartilage and bony ECMs. However, injectable hydrogels synthesized from natural biomaterials usually lack mechanical strength, thus limiting their potential utilization. In contrast, synthetic biomaterials-based injectable hydrogels have favorable stability and mechanical properties, but have poor biocompatibility and bioactive properties. Then, various preparation methods of injectable hydrogels, including both physical and chemical methods, were highlighted. Physical hydrogels can be easily fabricated, owing to their sensitivity to external stimuli such as temperature, pH, ion concentration, and stress. Although physical injectable hydrogels can easily be produced and have low cytotoxicity, they usually have a slow response time and low stability. In contrast, injectable hydrogels prepared via chemical methods show favorable stability under physiological conditions and excellent mechanical properties, but they have adverse effects *in vivo*, owing to chemical reactions.

Over the past several years, there have been many studies focused on synthesizing novel injectable hydrogels for cartilage and bone repair. However, many challenges remain to be addressed in fabricating injectable hydrogels to optimally achieve cartilage and bone regeneration. The major challenge of developing injectable hydrogels for cartilage and bone tissue engineering is the design of bioactive scaffolds that have perfect biocompatibility, biodegradability, stability, and favorable mechanical properties for 3D cell culture, and are able to support nutrient transportation and growth factor delivery. To address this challenge, first, bioactive biomaterials that can be used to prepare novel injectable hydrogels should be developed. Most recently, attempts at using glycopolypeptide,^291^ silk,^292^ carrageenan,^293^ pectin,^294^ and even the ECM^295^ to synthesize injectable hydrogels have attracted attention. Second, advanced fabrication methods require further development, primarily to improve the mechanical properties and physiological stability, and to decrease the cytotoxicity and adverse effects of the hydrogels *in vivo*. Finally, the development of a methodology to integrate the merits of the various biomaterials and fabrication methods for the preparation of injectable hydrogels will play an important role in the clinical applications of hydrogels in cartilage and bone tissue engineering.
